# Supplementary material for: AKR1B10, One of the Triggers of Cytokine Storm in SARS-CoV2 Severe Acute Respiratory Syndrome
Source: Int J Mol Sci. 2022 Feb 8;23(3):1911. doi: 10.3390/ijms23031911 (PMC8836815; doi:10.3390/ijms23031911)
Supplement: Supplementary file 1 [file ijms-23-01911-s001.zip › ijms-1584998-supplementary.pdf]

Supplemental Data

AKR1B10, One of the Triggers of Cytokine Storm in SARS-CoV2 Severe Acute Respiratory Syndrome

Clovis Chabert [1,*], Anne-Laure Vitte [1], Domenico Iuso [1], Florent Chuffart [1], Candice Trocme [2], Marlyse Buisson [3], Pascal Poignard [3], Benjamin Lardinois [4], Régis Debois [4], Sophie Rousseaux [1], Jean-Louis Pepin [5,6], Jean-Benoit Martinot [7,8] and Saadi Khochbin [1]

[1] Institute for Advanced Biosciences—UGA—INSERM U1209—CNRS UMR 5309, 38700 La Tronche, France; anne-laure.vitte@univ-grenoble-alpes.fr (A.-L.V.); domenico.iuso@univ-grenoble-alpes.fr (D.I.); florent.chuffart@univ-grenoble-alpes.fr (F.C.); sophie.rousseaux@univ-grenoble-alpes.fr (S.R.); saadi.khochbin@univ-grenoble-alpes.fr (S.K.)
[2] Laboratoire BEP (Biochimie des Enzymes et les Protéines), Institut de Biologie et de Pathologie, CHU Grenoble Alpes, 38700 La Tronche, France; ctrocme@chu-grenoble.fr
[3] Institut de Biologie Structurale, CEA, CNRS and Centre Hospitalier Universitaire Grenoble Alpes, Université Grenoble Alpes, 38000 Grenoble, France; marlyse.buisson@ibs.fr (M.B.); pascal.poignard@ibs.fr (P.P.)
[4] Laboratory Department, CHU UCL Namur Site de Ste Elisabeth, 5000 Namur, Belgium; benjamin.lardinois1@gmail.com (B.L.); regis.debois@uclouvain.be (R.D.)
[5] HP2 Laboratory, INSERM U1300, Grenoble Alpes University, 38000 Grenoble, France; JPepin@chu-grenoble.fr
[6] Sleep Laboratory, Pole Thorax et Vaisseaux, Grenoble Alpes University Hospital, 38000 Grenoble, France
[7] Sleep Laboratory and Pulmonology and Allergy Department—CHU UCL Namur, St. Elisabeth Site, 5000 Namur, Belgium; martinot.j@respisom.be
[8] Institute of Experimental and Clinical Research, UCL Bruxelles Woluwe, 1200 Brussels, Belgium
* Correspondence: mailto:clovis.chabert@inserm.fr; Tel.: +33-6-8898-4506

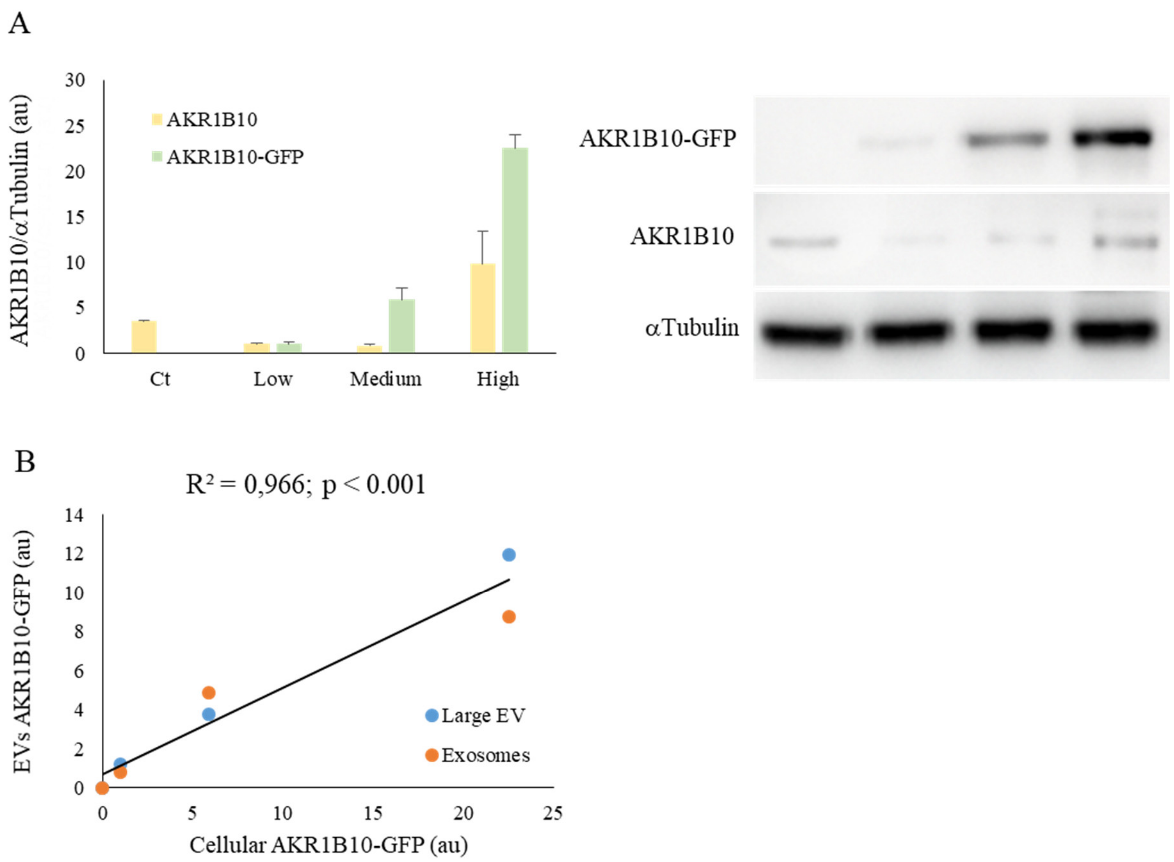

Figure S1. Increase of AKR1B10 endogenous and AKR1B10GFP in H1299 permanently transfected cells lines sorted by FACS. (A) transfected H1299 sorted according to quantile of GFP signal; (B) correlation between AKR1B10GFP cells expression and its concentration in Extracellular Vesicles. (n = 2-3; mean±SEM).

To test if the AKR1B10 proteins produced by lung cells may be transmitted to the macrophages *via* the Extracellular Vesicles (EVs), a pulmonary cells line (H1299) was transfected with a pEGFP-AKR1B10$_{GFP}$ plasmid, selected with antibiotics, and then sorted into 4 populations. EVs were extracted by successive ultra-centrifugations steps from the media of cell culture. Supplemental Figure S1A shows a clear increase of AKR1B10$_{GFP}$ level in the transfected cells and a sharp difference between the different H1299 cells lines that were selected by cell sorter (AKR1B10 in H1299$_{high}$ is 22 times higher than H1299$_{low}$ and 3.8 times higher than H1299$_{medium}$. H1299$_{high}$ is also associated with an increase of AKR1B10 endogenous, suggesting a positive regulation loop of the protein on its own expression. Interestingly, the concentration of AKR1B10 inside the cell is correlated to the quantity of the protein inside the EVs (R^2: 0.966; $p<0.001$).

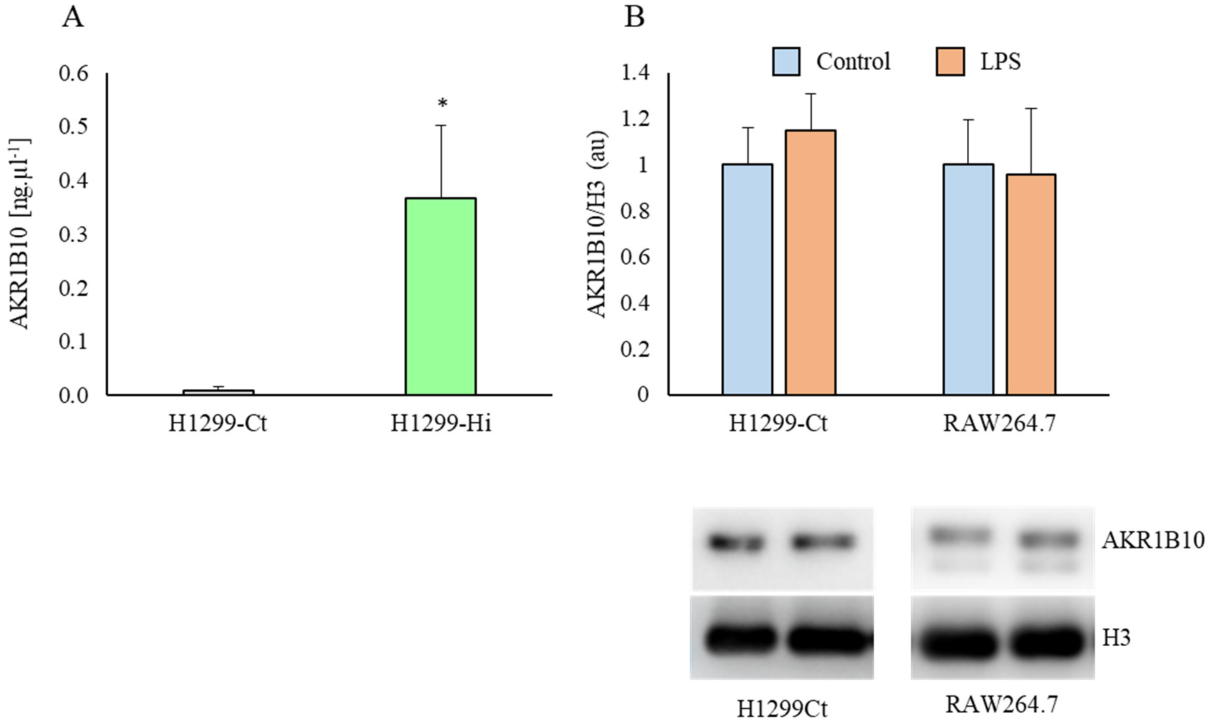

Figure S2. AKR1B10 concentrations in culture media of H1299 cells with (H1299-Ct) or without (H1299-Hi) AKR1B10-GFP transfection measured by ELISA (A). AKR1B10 production in H1299-Ct and RAW264.7 cells exposed to $1\mu g.ml^{-1}$ of LPS during 6h (B). (mean±SEM; n = 2-3); *: H1299-Hi diff. of H1299-Ct (*: $p<0.05$).

To test the amount of AKR1B10 protein released by H1299 in cell culture media, we collected the media after 24h of incubation and measured the concentration of the protein with the ELISA kit used for patients' blood samples (Cf. Fig. S2A). Our results highlight a sharp increase of AKR1B10 in the media of H1299 that overexpress AKR1B10, although the resulting concentration is far below the AKR1B10 concentrations measured in the sera of COVID-19 patients. Figure S2B represents the concentration of AKR1B10 in H1299 and RAW264.7 cells exposed to lipopolysaccharides during 6h at $1\mu g.ml^{-1}$ (Cf. Fig. S2B). The results show that AKR1B10 concentration in these cells does not change in our experimental conditions.

Supplementary Table S1. Sequences of primers used for RT-PCR on RAW264.7 and H1299 cells.

	Forward sequence	Reverse sequence
	**RAW264.7**	
TNFα	TTC-TGT-CTA-CTG-AAC-TTC-GGG-GTG-ATC-GGT-CC	GTA-TGA-GAT-AGC-AAA-TCG-GCT-GAC-GGT-GTG-GG
IL-6	AGG-ATA-CCA-CTC-CCA-ACA-GAC-CT	CAA-GTG-CAT-CAT-CGT-TGT-TCA-TAC
IL-1β	ATG-GCA-ACT-GTT-CCT-GAA-CTC-AAC-T	CAG-GAC-AGG-TAT-AGA-TTC-TTT-CCT-TT
GADPH	CAT-GGC-CTT-CCG-TGT-TCC-T	GCG-GCA-CGT-CAG-ATC-CA
Actin	CAT-GTA-CGT-TGC-TAT-CCA-GGC	CTC-CTT-AAT-GTC-ACG-CAC-GAT
	**H1299**	
TNFα	AGA-ACT-CAC-TGG-GGC-CTA-CA	AGG-AAG-CCC-TAA-GGT-CCA-CT
IL-6	TATG-AAC-TCC-TTC-TCC-ACA-AGC	GTT-TTC-TGC-CAG-TGC-CTC-TTT-G
IL-1β	AAT-CTG-TAC-CTG-TCC-TGC-GTG-TT	TGG-GTA-ATT-TTT-GGG-ATC-TAC-ACT-CT
U6	CTC-GTC-TCG-GCA-GCA-CA	AAC-GCT-TCA-CGA-ATT-TGC-GT
Actin	CAT-GTA-CGT-TGC-TAT-CCA-GGC	CTC-CTT-AAT-GTC-ACG-CAC-GAT

Supplemental Methods

1.1 *Cell culture*

H1299 cells were cultured in RPMI1640 media with 10% of foetal bovine serum and antibiotics (PenStrep 1X). All the transfections were performed with lipofectamine2000, Optimem, and a pEGFP-C2 vector containing the coding sequence of AKR1B10. Transfection were performed 24h before mRNA extraction in 6 wells plate with 200µl of Optimem, 1-3µg of plasmid and 3-9µL of Lipofectamine2000.

1.2 *Cells transfection*

H1299 transfection were performed in 1000mm well plate with 500µl of Optimem, 5µg of plasmid, and 15µL of Lipofectamine2000. During 2 weeks, the cells were cultured with 100µL of Neomycin to remove non-transfected cells and then sort with a cell sorter (BD FACS Aria IIu Cell Sorting System) according to the quantile level of GFP expression, which corresponds to $H1299_{Low}$, $H1299_{Medium}$, $H1299_{High}$.

1.3 *Western blotting*

Electrophoresis were performed on a Mini-Bio-Rad system with precast gradient gel 8-20% and the transfer on nitrocellulose membrane 0.4µm were accomplished in the FastTurbo blot machine (Bio-Rad). The antibody used for quantification were: AKR1B10 (Ab96417); α-tubulin (T5168).
